# Fine-scale spatial distribution of deltamethrin resistance and population structure of *Anopheles funestus* and *Anopheles arabiensis* populations in Southern Mozambique

**DOI:** 10.1186/s12936-023-04522-5

**Published:** 2023-03-14

**Authors:** Smita Das, Mara Máquina, Keeley Phillips, Nelson Cuamba, Dulcisaria Marrenjo, Francisco Saúte, Krijn P. Paaijmans, Silvie Huijben

**Affiliations:** 1 The Center for Evolution & Medicine, School of Life Sciences, Arizona State University, Tempe, AZ USA; 2 Centro de Investigação em Saúde de Manhiça (CISM), Fundação Manhiça, Manhica, Mozambique; 3 Programa Nacional de Controlo da Malária, Ministério da Saúde, Maputo, Mozambique; 4 PMI VectorLink Project, Abt Associates Inc., Maputo, Mozambique; 5 Simon A. Levin Mathematical, Computational and Modeling Sciences Center, Arizona State University, Tempe, AZ USA; 6 The Biodesign Center for Immunotherapy, Vaccines and Virotherapy, Arizona State University, Tempe, AZ USA; 7 Present Address: PATH, Seattle, WA USA; 8 ISGlobal, Barcelona, Spain

**Keywords:** Population genetics, Malaria, Insecticide resistance, Surveillance, *Anopheles gambiae*, *Anopheles funestus*, Gene flow

## Abstract

**Background:**

Insecticide resistance in malaria vectors can be spatially highly heterogeneous, yet population structure analyses frequently find relatively high levels of gene flow among mosquito populations. Few studies have contemporaneously assessed phenotypic, genotypic and population structure analysis on mosquito populations and none at fine geographical scales. In this study, genetic diversity, population structure, and insecticide resistance profiles of *Anopheles funestus* and *Anopheles arabiensis* were examined across mosquito populations from and within neighbouring villages.

**Methods:**

Mosquitoes were collected from 11 towns in southern Mozambique, as well as from different neighbourhoods within the town of Palmeira, during the peak malaria transmission season in 2016. CDC bottle bioassay and PCR assays were performed with *Anopheles* mosquitoes at each site to determine phenotypic and molecular insecticide resistance profiles, respectively. Microsatellite analysis was conducted on a subsample of mosquitoes to estimate genetic diversity and population structure.

**Results:**

Phenotypic insecticide resistance to deltamethrin was observed in *An. funestus *sensu stricto (*s.s.*) throughout the area, though a high level of mortality variation was seen. However, 98% of *An. funestus s.s.* were *CYP6P9a* homozygous resistant. *An. arabiensis* was phenotypically susceptible to deltamethrin and 99% were *kdr* homozygous susceptible. Both *Anopheles* species exhibited high allelic richness and heterozygosity. Significant deviations from Hardy–Weinberg equilibrium were observed, and high linkage disequilibrium was seen for *An. funestus s.s.,* supporting population subdivision. However, the F_ST_ values were low for both anophelines (− 0.00457 to 0.04213), N_m_ values were high (9.4–71.8 migrants per generation), AMOVA results showed almost 100% genetic variation among and within individuals, and *Structure* analysis showed no clustering of *An. funestus s.s.* and *An. arabiensis* populations. These results suggest high gene flow among mosquito populations.

**Conclusion:**

Despite a relatively high level of phenotypic variation in the *An. funestus* population, molecular analysis shows the population is admixed. These data indicate that *CYP6P9a* resistance markers do not capture all phenotypic variation in the area, but also that resistance genes of high impact are likely to easily spread in the area. Conversely, other strategies, such as transgenic mosquito release programmes will likely not face challenges in this locality.

**Supplementary Information:**

The online version contains supplementary material available at 10.1186/s12936-023-04522-5.

## Background

With more than 600,000 malaria deaths worldwide in 2020, malaria continues to be a major problem in endemic tropical regions [1]. Vector control is one of the most effective interventions to reduce and prevent the spread of the disease, as was evidenced by the huge reductions in cases following the large-scale distributions of long-lasting insecticidal bed nets (LLINs) and indoor residual spraying (IRS) since the start of this century [2]. However, the successes over the past two decades have not continued, and for the past 5 years a plateau in the annual number of cases has been observed, and more recently an increase due to the COVID-19 pandemic [1]. The widespread distribution of insecticide-resistant mosquitoes, particularly against pyrethroids—the most used active ingredient in LLINs—is likely one of the main contributors to this observed plateau [1, 3]. To prevent the emergence and spread of resistance to new active ingredients that are in development, and reduce the selection pressure against currently available insecticides, resistant management strategies (RMS) are required. These include approaches such as insecticide combination, rotation, or mosaic applications, though there are many challenges in the implementation of such strategies [4–6]. To understand which of the different RMS reduce the selection for insecticide resistant mosquitoes most effectively, and whether this is location- and/or species-specific, it is important to understand the evolutionary ecology of malaria mosquitoes.

Resistance evolution is driven by the random appearance of a first de novo mutant, and the subsequent selection acting on this mutant in the presence of the insecticide [7]. The relative importance of each of these processes and the rate of spread is not yet understood and will depend on many organism-specific factors such as mutation rates, the level of genetic variation already present in the population, insecticide pressure, fitness costs, and gene flow. Insecticide resistance is typically monitored across several sentinel sites in a country and spatial variation in insecticide resistance for *Anopheles* species is frequently observed between populations from different regions of the same country [8–11]. However, significant variation in insecticide resistance, both at the phenotypic and genotypic level, has also been observed at much finer spatial scale, such as in neighbouring villages, as detected for *An. arabiensis* in south-eastern Tanzania [12] and for *Aedes aegypti* even at the city block level [13, 14]. It is not clear whether such spatial heterogeneity is predominantly driven by complex evolutionary dynamics of frequent (re)introduction of resistance genes through mutations and/or gene flow, followed by local selection against them, or the result of limited or no gene flow [9, 15, 16].

Many population genetic studies have shown that over large geographical space (across one or multiple countries), mosquito populations fall in different clusters with somewhat limited gene flow, as seen for *An. arabiensis* [17, 18], *An. funestus* [9, 19–22], *Anopheles gambiae* [23] and *Anopheles coluzzii* [23]. On a smaller geographic scale, genetic differentiation is not always found (e.g. for *An. gambiae s.s.* [24], *An. arabiensis* [25], and *An. funestus s.s* [20]). However, under certain conditions genetic differentiation may exist between geographically close locations, for instance for *An. arabiensis* populations in the south of Tanzania [24] and in east Sudan [18], likely due to ecologically diverse environments. In Mozambique, population differentiation of *An. arabiensis* were observed in villages less than 25 km apart in an area consisting of a mosaic of suitable habitats and thus a higher likelihood of discreet populations subjected to genetic drift [17]. Further, genetically different nearby *Anopheles stephensi* populations were observed in eastern Ethiopia, likely due to independent introductions from southern Asia [26]. Lastly, and perhaps less surprisingly, *An. gambiae s.s.* populations on various Lake Victoria islands that are separated by 4–50 km with each other and mainland Uganda, have also been found to be genetically differentiated from each other [27]. Yet, a historical genetic sweep of resistance gene *cyp6p* was detected across these island populations, demonstrating that some gene flow occurred between the islands and the mainland. Overall, it is not necessarily physical barriers (except vast geographical barriers such as the Rift Valley) or geographic distance, but other factors that drive gene flow, such as climate, ecosystem, or cultural practices [28].

However, few studies combine insecticide resistance data, on both the phenotypic and genotypic scale, with population structure analysis, particularly at a fine geographic scale. Evaluating the complex genetic diversity of mosquito vectors among and within populations can be a powerful tool for understanding gene flow and the likelihood of the spread of genes that confer insecticide resistance. In combination with insecticide resistance monitoring data, population genetic studies can support malaria programmes in making evidence-based, targeted programmatic decisions for IRS and LLIN selection and the deployment of novel vector control strategies (such as future gene-drive approaches). The level of gene flow is an essential parameter for the design of spatial insecticide application strategies, such as mosaic treatments (the spatial distribution of two insecticides with unique active ingredients) to determine the level at which mosaic treatments should be designed. For example, in areas of low gene flow, the mosaic will need to be designed at a much finer scale level than in areas of high gene flow, since otherwise mosquito generations will predominantly be exposed to monotreatment. Gene drive mosquitoes are currently being tested for population suppression systems in their ability to either drive the mosquito population to extinction [29, 30] or to replace the population with one that is refractory for malaria transmission [31, 32]. A sufficiently high level of gene flow is needed for the successful spread of the gene through the population, or many spatially distributed releases would be needed [27, 33, 34].

Here, we concurrently investigate the population structures and insecticide resistance profiles of *An. arabiensis* and *An. funestus s.s.* in 11 towns within a 1650 km^2^ area in Manhiça district, and parts of Magude district and Bilene district in southern Mozambique. The town of Palmeira has previous been identified as containing a population of *An. funestus s.l.* mosquitoes that are highly resistant to pyrethroids [35, 36]. It is unclear what the distribution of insecticide resistance is in the neighbouring villages of Palmeira. Additionally, *An. funestus* population structure on fine spatial scale has yet to be established [17]. Such analysis can be used to anticipate the spread of genes that confer insecticide resistance and consider novel approaches toward reaching malaria elimination in southern Mozambique.

## Methods

### Mosquito collection

**Fig. 1:**
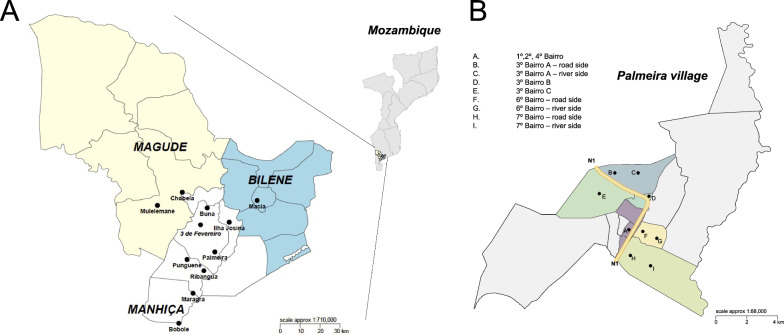
Map of study area in southern Mozambique **A** showing the eight sampled villages in the district of Manhiça, two in the district of Magude and one in the district of Bilene and **B** showing the eight neighbourhoods within the village of Palmeira. National highway N1 that runs through the village is shown in yellow with orange borders. A river runs along the east border of the village boundaries

From March 9 to May 4, 2016, during peak mosquito and malaria transmission season toward the end of the rainy season, *Anopheles* mosquitoes were collected in a large variety of houses across 11 towns in Maputo Province in Southern Mozambique: Bobole, Buna, Chobela, Ilha Josina, Macia, Magude (Mulelemani), Maragra, Palmeira, Punguene, Ribangua, and 3 de Fevereiro (Fig. 1A), with a minimum of ten houses visited at each site. In the village of Palmeira, additional sampling was performed on the neighbourhood level (Fig. 1B). Female anopheline mosquitoes were collected indoors during the early morning (5:00–8:00 AM), using a handheld mouth aspirator and torch and transported in paper cups to the laboratory. All mosquitoes were morphologically identified using dichotomous keys for *Anopheles* in southern Africa [37, 38].

Mosquitoes were tested within an hour of arrival for insecticide resistance to 1X or 5X deltamethrin (10 and 50 μg/bottle respectively) using the CDC bottle bioassay following the guidelines [39]. Mortality was recorded at 15-min intervals, with mortality at 30 min used as the diagnostic time. At the end of the two-hour exposure, dead and knocked down mosquitoes were separated from those alive, killed and stored individually in tubes with silica gel. If multiple tests were performed on the same collection day, the same control bottles were shared (i.e. same controls for exposure to both 1X and 5X deltamethrin; up to four exposure bottles per control bottle). If control mortality was between 3 and 10%, the Abbott’s formula was applied to correct for background mortality [39]. If control mortality was higher than 10%, the test was discarded and a new test was performed [37, 38]. At least 30 individuals of each species complex, incl. *An. gambiae *sensu lato (*s.l*.), and group (*An. funestus s.l.*) from each sampling site, when available, were selected for the population genetics component in this study based on previous work by Hale and colleagues who showed that 25–30 individuals per population are adequate for microsatellite-based studies [40].

### DNA extraction and species identification

For each collected female anopheline, the head and thorax were separated from the abdomen. DNA was extracted from the abdomen using a commercial DNA extraction kit (DNeasy Blood and Tissue Kit, Qiagen, Hilden, Germany). Polymerase chain reaction (PCR) for specific members of the *An. gambiae* or *An. funestus* complexes were performed to confirm the morphological identifications [41, 42]. If the assays did not identify the species, then another PCR that amplifies the ITS2 gene and detects a range of African *Anopheles* mosquito species was used to determine species [42].

### Insecticide resistance molecular detection

For *An. gambiae s.l.*, the PCRs for detection of the east (leucine to serine substitution; L1014S) and west (leucine to phenylalanine substitution; L1014F) mutations of the knock down resistance (*kdr*) gene for pyrethroid resistance were performed as previously described [43, 44]. The *kdr* mutation affects the voltage-gated sodium channel in *Anopheles* mosquitoes and has been identified in *An. gambiae s.s* [43, 45] and *An. arabiensis* [46, 47]. For *An. funestus s.l.*, a restriction fragment length polymorphism PCR (RFLP-PCR) assay was used to identify a major resistance gene *CYP6P9a* that confers cytochrome P450-mediated resistance to pyrethroid insecticides [48]. This PCR amplifies a partial *CYP6P9a* upstream region containing a restriction site which, if present, is then digested by TaqI restriction enzyme using RFLP assay [48].

### Microsatellite DNA amplification

Primers for fourteen *An. gambiae s.s.* microsatellite loci were screened [49], of which six were chosen based on consistent amplification and detection of the loci by sequencing in both *An. gambiae s.s.* and *An. arabiensis*. These six loci provide are distributed relatively evenly throughout the genome, providing good coverage: *AGXH25* and *AGXH100* on the X chromosome, *AG2H85* and *AG2H164* on chromosome 2, and *AG3H127 and AG3H249* on chromosome 3. Each mosquito sample was tested for all six microsatellites by PCR amplification followed by capillary electrophoresis fragment analysis [24]. For each mosquito, two multiplex PCRs were done, each differing only in the primer mix; primer mix 1 contained primers for *AGXH25*, *AG2H85*, *AG2H164* loci and primer mix 2 contained primers for *AG3H127, AGXH100, AG3H249* loci (Additional file 1: Table S9). The forward primer in each reaction was labeled with fluorescent marker HEX or FAM. The primer mix for each PCR was made to a final volume of 400 µL and 0.5 µM concentration of all primers (Integrated DNA Technologies, Coralville, Iowa). Each 25 µL PCR reaction consisted of 12.5 µL Platinum™ Multiplex PCR Master Mix (Applied Biosystems, Foster City, California), 5 µL primer mix, 2 µL DNA template, and 5.5 µL RNase free water. If amplification of one or more loci was not detected, then each locus would be re-run separately. The primer mix for each multiplex set was made to a final volume of 400 µL and 0.5 µM concentration of all primers. The forward primer in each reaction was labeled with fluorescent marker HEX or FAM. The PCR was performed with an initial 5 min denaturation at 95 °C, followed by 29 cycles of 20 s at 95 °C, 30 s at 55 °C, and 30 s at 72 °C. The final incubation step was 1 h at 72 °C. If amplification of one or more loci in a multiplex reaction was not detected, then each locus would be re-run separately.

For *An. funestus s.l.*, 13 microsatellites were selected from published sequence data [50–53] that indicated high polymorphism and no evidence for null alleles. Of the 13 microsatellites, six were selected based on consistent amplification and detection by sequencing and distribution in genome, though it should be noted that none of the selected microsatellites are located on the X chromosome: *FunO, AFND32, AFND40, AFND6* on chromosome 2, and *FunG* and *FunD* on chromosome 3. Each mosquito sample was tested for all six microsatellites by PCR amplification followed by capillary electrophoresis fragment analysis. Microsatellites were amplified by PCR in two multiplex reactions containing the primers for the following loci: (1) *FunO*, *FunG*, *AFND6* and (2) *AFND32*, *FunD*, *AFND40* (Additional file 1: Table S9). The forward primer in each reaction was labeled with fluorescent marker HEX or FAM (Additional file 1: Table S9). Each 25 µL reaction for each mosquito consisted of 2.5 µL 10X reaction buffer without MgCl_2_, 1.5 µL 25 mM MgCl_2_, 0.5 µL dNTPs (200 µM each), 0.1 µL forward and reverse primers (100 µM each; Integrated DNA Technologies, Coralville, Iowa), 0.2 µL (1 U) Taq polymerase, 2 µL DNA template, and 17.7 µL RNase free water. The PCR was performed with an initial denaturation of 2 min at 94 °C, followed by 34 cycles of 30 s at 94 °C, 30 s at 54 °C, and 30 s at 72 °C, and then a final incubation step of 10 min at 72 °C. If amplification of one or more loci in a multiplex reaction was not detected, then each locus would be re-run separately.

For both *An. gambiae s.l.* and *An. funestus s.l.* microsatellite amplification, 1 µL of each PCR product was mixed with GeneScan™ 500 ROX size standard (Perkin-Elmer, Norwalk, Connecticut) and deionized formamide as directed by the manufacturer, and then run on the ABI 3730 Genetic analyzer. Alleles were identified by using the ABI 3730 Genemapper (Applied Biosystems, Foster City, California) software.

### Statistical analysis and microsatellite allele frequencies

#### Insecticide resistance

For each locality, knock-down mortality at 30 min exposure (diagnostic time) and 120 min exposure (end of test) is reported. Cumulative mortality was calculated for localities where mosquitoes were tested over multiple days due to the low numbers of mosquitoes collected daily. Following morphological identification, 120-min mortality was calculated for *An. funestus* and *An. gambiae s.l.* separately, as well as for *Anopheles rufipes* that was abundant at one location (Chobela). Mortality data are reported by village level (Palmeira neighbourhoods (1°, 2° and 4° Bairro representing the village Palmeira) and by neighbourhood level for Palmeira. Chi-square tests were used to estimate 95% confidence intervals for mortality.

#### Genetic diversity and population structure

Genetic diversity of microsatellite alleles was investigated using *Arlequin* 3.5.2.2 [54]. Mosquitoes that failed to amplify at ≥ 1 microsatellite loci were excluded from analysis. Loci, both individually and by population were tested for significant deviation from Hardy–Weinberg equilibrium using a Markov-chain algorithm with 1,000,000 steps and 100,000 dememorization steps [55] and further assessed by the overall inbreeding coefficient (F_IS_) [56]. Pairs of microsatellite loci were assessed for linkage disequilibrium using a test procedure analogous to Fisher’s exact test and set to 10,000 permutations [18, 57]. To account for multiple comparisons, significance threshold was adjusted using the Šidák correction method, $1-{(1-0.05)}^{(\frac{1}{n})}$ where n is the number of independent comparisons [58, 59]. *Arlequin* was also used to perform locus by locus analysis of molecular variance (AMOVA) to determine the contribution of genetic variation within and among populations of both mosquito species to the overall genetic diversity, as well as to investigate pairwise F_ST_ values. The significance of F_ST_ distance was determined by 10,000 permutations [24, 60–63]. Gene flow was estimated by determining the number of migrants per population per generation, also known as N_m_, and was calculated using the equation, ${\mathrm{N}}_{\mathrm{m}}=(1-{F}_{ST})/4{F}_{ST}$. *Structure* was used to apply a Bayesian model-based clustering algorithm to characterize population structure and designate mosquitoes to pre-determined clusters (*K*) based on individual multilocus genotype data [64]. The distinct number of clusters in the data set (*K*) was estimated from 1 to 5 for *An. gambiae s.l.* and 1–7 for An*. funestus s.l.* by the posterior log probability data under each *K*, Ln [Pr(*X*|*K*)]. Five replicates were performed per *K* clusters. Using an admixture model and correlated allele frequencies, each run was carried out with 1,000,000 iterations after a burn-in period of 100,000 [22]. The estimated number of clusters in the study population was decided by the *K* value with the highest Ln [Pr (X|K)].

## Results

**Fig. 2:**
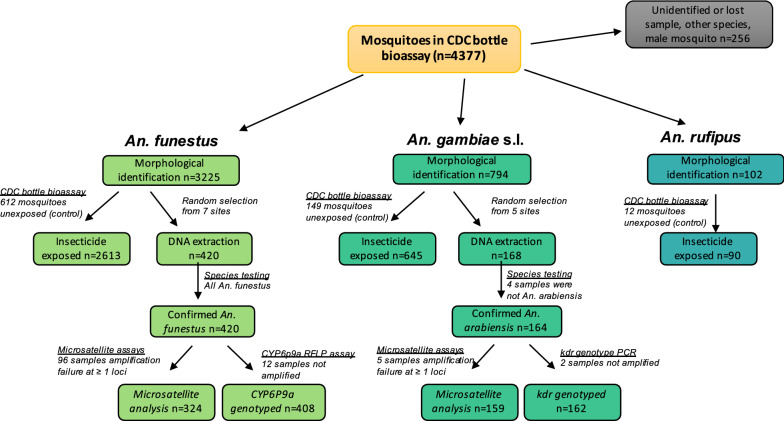
Flow chart of number of mosquito samples at analytical steps in the project

A total of 4377 mosquitoes were collected from the different study sites and tested in the bottle bioassays (Fig. 2). Of these, 3225 were morphologically identified as *An. funestus s.l.*, 794 as *An. gambiae s.l.*, 102 as *An. rufipes,* 1 as *Anopheles. tenebrosus*, and 1 as *Anopheles pharoensis*. A total of 254 mosquitoes (5.8%) were excluded for a variety of reasons, including they were missing from the tube, the mosquito was damaged, the tube was wrongly or poorly labeled, a male mosquito was collected, or because a mosquito was identified as other than *An. funestus s.l., An. gambiae s.l*. or *An. rufipes*. A total of 168 *An. gambiae s.l.* and 420 *An. funestus s.l.* were selected from study sites where at least 30 mosquitoes of each species complex (*An. gambiae s.l*.) and group (*An. funestus s.l*.) were available for further molecular analysis. Of these, *An. gambiae s.l.* mosquitoes were from 5 study sites (Fig. 1): Punguene, Magude (Mulelemane), Chobela, Ilha Josina, and Palmeira (from all neighbourhoods). *Anopheles funestus s.l.* mosquitoes were collected from 7 study sites (Fig. 1): Punguene, Ribangua, 3 de Fevereiro, Bobole, Macia, and 4 neighbourhoods in Palmeira (1°, 2°, 4° and 7° Bairro). Of the 168 *An. gambiae s.l.*, 164 were confirmed as *An. arabiensis* and the remaining 4 mosquitoes were composed of 1 *Anopheles leesoni*, 2 *An. funestus s.s.*, and 1 undetermined. Of the 420 *An. funestus s.l.,* all were confirmed as *An. funestus s.s.*. Hereafter, we refer to morphologically identified *An. gambiae s.l.* as *An. arabiensis* and *An. funestus s.l. as An. funestus s.s.*. In total, 164 *An. arabiensis* and 420 *An. funestus s.s.* were screened for 6 respective microsatellite loci. Five *An. arabiensis* mosquitoes were excluded based on the amplification failure criteria of ≥ 1 loci, resulting in 159 mosquitoes for analysis. Amplification failure at ≥ 1 loci occurred in 96 *An. funestus s.s.* mosquitoes and were excluded, resulting in 324 *An. funestus s.s.* mosquitoes that were used in the analysis. The highest rates of amplification failure occurred at the *AG3H249* locus for *An. arabiensis*, and the FunO and AFND6 loci for *An. funestus s.s.*

### Insecticide resistance

#### Level of phenotypic resistance

**Fig. 3:**
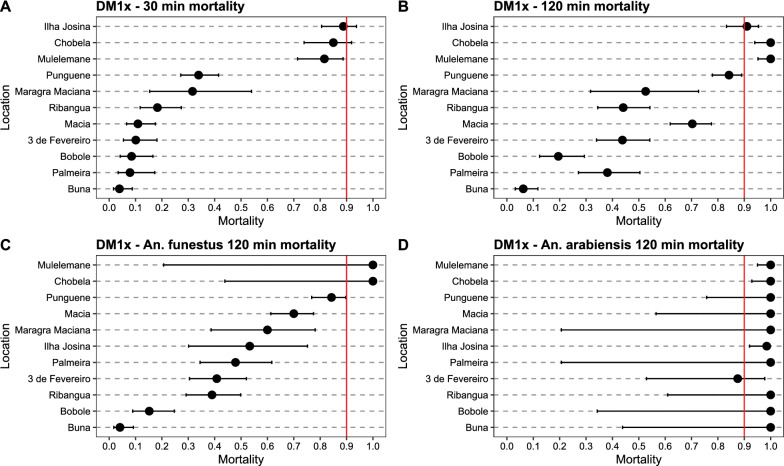
Deltamethrin 1 × CDC bottle bioassay mortality after a 30 min (**A**) and 120 min (**B**) exposure of collected populations across the district and species-specific mortality after 120 min for *An. funestus* (**C**) and *An. arabiensis* (**D**). Dots demonstrate proportion of mortality with error bars showing 95% confidence interval using Chi-square estimates. Red vertical line shows WHO cutoff value of 90% mortality, below which a population is determined resistant after a 30 min exposure. Locations are sorted by mortality in 30 min exposure in panels A&B and by *An. funestus* 120 min mortality in panels C&D. Palmeira data is from neighbourhood 1°/2°/4° Bairro

**Fig. 4:**
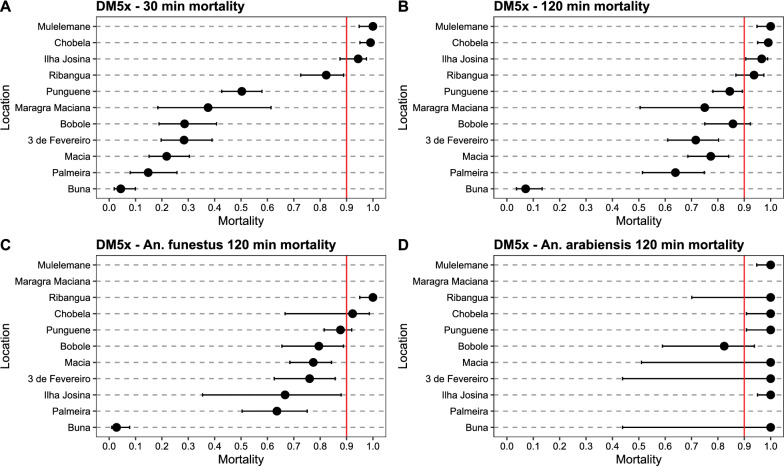
Deltamethrin 5 × CDC bottle bioassay mortality after a 30 min (**A**) and 120 min (**B**) exposure of collected populations across the district and species-specific mortality after 120 min for *An. funestus s.s.* (**C**) and *An. arabiensis* (**D**). Dots demonstrate proportion of mortality with error bars showing 95% confidence interval using Chi-square estimates. Red vertical line shows WHO cutoff value of 90% mortality, below which a population is determined resistant after a 30 min exposure. Locations are sorted by mortality in 30 min exposure in panels A&B and by *An. funestus s.s.* 120 min mortality in panels C&D. Palmeira data is from neighbourhood 1°/2°/4° Bairro

**Fig. 5:**
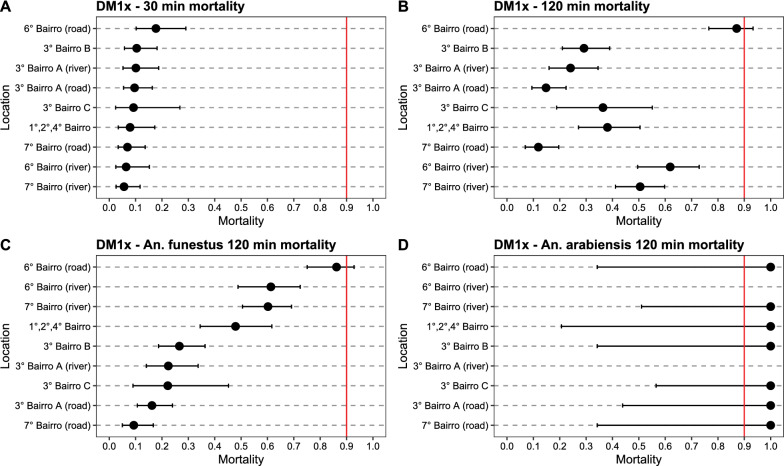
Deltamethrin 1 × CDC bottle bioassay mortality data after a 30 min (**A**) and 120 min (**B**) exposure of collected populations across the Palmeira neighborhoods and species-specific mortality after 120 min for *An. funestus s.s.* (**C**) and *An. arabiensis* (**D**). Dots demonstrate proportion of mortality with error bars showing 95% confidence interval using Chi-square estimates. Red vertical line shows WHO cutoff value of 90% mortality, below which a population is determined resistant after a 30 min exposure. Locations are sorted by mortality in 30 min exposure in panels A&B and by *An. funestus s.s.* 120 min mortality in panels C&D

**Fig. 6:**
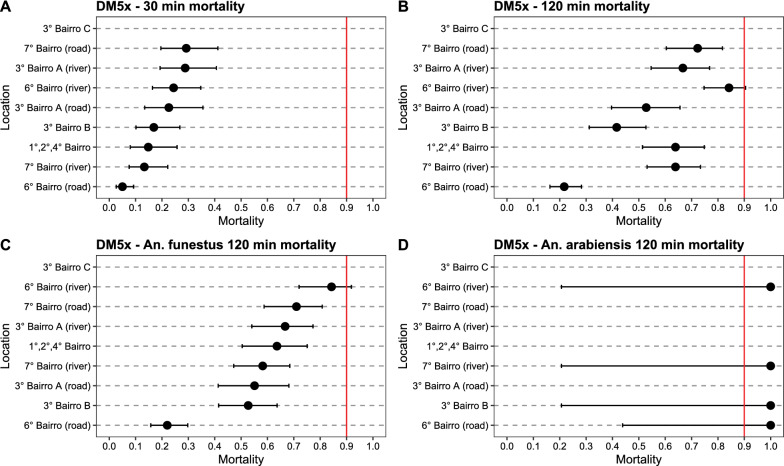
Deltamethrin 5 × CDC bottle bioassay mortality data after a 30 min (**A**) and 120 min (**B**) exposure of collected populations across the Palmeira and species-specific mortality after 120 min for *An. funestus s.s.* (**C**) and *An. arabiensis* (**D**). Dots demonstrate proportion of mortality with error bars showing 95% confidence interval using Chi-square estimates. Red vertical line shows WHO cutoff value of 90% mortality, below which a population is determined resistant after a 30 min exposure. Locations are sorted by mortality in 30 min exposure in panels A&B and by *An. funestus s.s.* 120 min mortality in **C**, **D**

All mosquito populations were determined resistant to deltamethrin, with 30-min mortality rates at 1 × diagnostic dose ranging from 3.9% (Buna) to 88.8% (Ilha Josina) (Additional file 1: Table S1, Fig. 3A). Mortality after 2-h exposure (associated with resistance intensity, see [65]) ranged from 6.2% (Buna) to 100% (Chobela) (Fig. 3B). Mortality at 30 min against 5 × diagnostic dose of deltamethrin similarly ranged from 4.4% (Buna) to 100% (Mulelemane) (Fig. 4A). Mosquitoes collected from all neighbourhoods in Palmeira were highly resistant against deltamethrin, with 30-min mortality at 1 × diagnostic dose ranging from 5.6% to 17.7% (Fig. 5) and at 5 × diagnostic dose being 5.0–29.2% (Additional file 1: Table S2, Fig. 6). Bendiocarb resistance was not observed in any of the four sites that were assayed (Chobela, Mulelemane, Palmeira and Ribangua) with 30-min mortality ranging from 99–100% at these sites (Additional file 1: Table S3).

Differences in 30-min mortality between sites were in part explained by differences in species composition. For example, 98% of mosquitoes collected from Buna (30-min mortality at 1 × deltamethrin being 3.9%) were *An. funestus s.s.*, whereas 96% and 99% of mosquitoes collected from Chobela and Mulelemane respectively (85.0 and 81.6% mortality, respectively, for 30-min exposure to 1 × deltamethrin) were *An. arabiensis* (Additional file 1: Table S1). Nearly all mosquitoes from Palmeira were identified as *An. funestus s.s.* (98%).

Species-specific mortality rates were only available at the 2-h exposure mark, since the CDC bottle bioassay was run for the full 2-h exposure, and mosquitoes were killed and stored at this timepoint for future species identification. Significant differences in susceptibility were observed among the *An. funestus s.s.* populations across space (Figs. 3C, 4C, Additional file 1: Figs. S1, S2). The 2-h mortality of localities with sample size of over 50 *An. funestus s.s.* assayed ranged from 4.1% (Buna) to 84.3% (Punguene). Even within the neighbourhoods of Palmeira, significant diversity was observed in 2-h mortality of the *An. funestus* population, ranging from 9.3% to 86.2%. In addition to *An. funestus s.s.* and *An. gambiae s.l.*, *An. rufipes* was collected, with 99 out of the 102 identified originating from Chobela. Of these, all were dead at the 120-min exposure mark in the different exposures: 9 in the 1 × deltamethrin exposure, 51 in the 5 × deltamethrin exposure, and 28 in the 1 × bendiocarb exposure (remaining *An. rufipus* were identified from control bottles).

#### Molecular resistance

Of the 164 *An. arabiensis* that were tested for the 1014 locus of the *kdr* gene that confers pyrethroid resistance, 162 (98.8%) were homozygous susceptible and 2 (1.2%) did not amplify. This is consistent with the 100% phenotypic susceptibility to 1X deltamethrin that was observed in this species across study sites, except for 3 de Fevereiro with 87.5% mortality. Of the 420 *An. funestus s.s.* mosquitoes, 408 had enough DNA extract available for the *CYP6P9a* gene detection PCR for pyrethroid resistance. 401 (98.3%) were homozygous resistant, one was heterozygous resistant (0.2%), two were homozygous susceptible (0.5%), and four (1.0%) did not amplify.

### Genetic variability

Genotypes of 159 *An. arabiensis* mosquitoes were analysed across 6 microsatellite loci. All microsatellite loci were polymorphic, of which the number of distinct alleles per locus ranged from 2 (*AG3H27*) to 18 (*AG3H249*), with a mean of 8.5 (Additional file 1: Table S4). It should be noted that AG3H127 was monomorphic in *An. arabiensis* collected from all study sites, except for Ilha Josina. The mean expected heterozygosity and observed heterozygosity were 0.55 and 0.38 respectively. A total of 324 *An. funestus s.s.* were analysed for polymorphic loci at 6 microsatellites. All *An. funestus s.s.* microsatellite loci were polymorphic and the number of alleles per locus ranged from 9 (AFND40) to 30 (FunD), with a mean of 15.8 (Additional file 1: Table S5). The mean expected heterozygosity and observed heterozygosity for *An. funestus s.s.* were 0.81 and 0.53, respectively.

### Hardy–Weinberg and linkage disequilibrium

When all *An. arabiensis* mosquitoes from the five populations were pooled and analysed as one group, four loci (*AGXH25*, *AG2H85, AG3H127, AG3H249*) had significant heterozygote deficiency and did not conform to Hardy–Weinberg equilibrium (P < 0.000197) (Additional file 1: Table S4). Within each of the five populations, significant deviation from Hardy–Weinberg was also observed in locus *AGXH25*, *AG2H85*, and *AG3H127* (Additional file 1: Table S4). Similarly, *An. funestus s.s.* mosquitoes from seven populations were combined and analysed as one group, resulting in significant deviations from Hardy–Weinberg across all loci (P < 0.00122) (Additional file 1: Table S5). Hardy–Weinberg was violated across most loci within each of the seven populations (P < 0.00122). The heterozygote deficiency observed for both *An. arabiensis* and *An. funestus s.s.* suggests admixture or subdivision within respective populations.

Linkage disequilibrium describes the non-random association of different alleles of loci in a population. In an ideal population where forces such as genetic drift, selection, and inbreeding are absent, linkage disequilibrium should approach zero [66, 67]. None of the overall pairwise comparisons among loci in *An. arabiensis* showed linkage disequilibrium (P > 0.00341). Within populations, only *An. arabiensis* from Ilha Josina had one significant overall pairwise comparison (P < 0.00341), *AG2H164* and *AGXH100*. The low range of linkage disequilibrium across populations of *An. arabiensis* indicates no detectable population subdivision. In contrast, for *An. funestus s.s.,* five (33.3%) of the overall pairwise comparisons were statistically significant (P < 0.00341, Additional file 1: Table S6), suggesting that population subdivision may be present. Within populations, linkage disequilibrium was low ranging from 0% to 13.3% of significant pairwise comparisons (P < 0.00341) suggesting little to no substructure within each collection site.

### Population structure

The amount of genetic differentiation among populations is referred to as the F_ST_ estimate. For *An. arabiensis*, F_ST_ values between pairs of populations were low, ranging from 0.00443 to 0.0259, and none were significant (P > 0.05) (Table 1a). Overall, the F_ST_ values indicate little genetic differentiation among *An. arabiensis* populations. The pairwise population F_ST_ estimates for *An. funestus s.s.* ranged from − 0.00457 to 0.04213. Four of the *An. funestus s.s.* pairwise F_ST_ estimates were statistically significant (Table 1b). Like *An. arabiensis*, the low Fst values suggest that the *An. funestus s.s.* populations are mostly interbreeding freely.

**Table 1:** Estimates of F_ST_ values for *An. arabiensis* and *An. funestus*

a)* An. arabiensis*					
Populations	Punguene	Magude-Mulelemani	Palmeira 1°/2°/4° Bairro	Chobela	Ilha Josina
Punguene	*				
Magude-Mulelemani	0.00526	*			
Palmeira 1°/2°/4° Bairro	0.01454	0.00999	*		
Chobela	0.00936	0.00815	0.01045	*	
Ilha Josina	0.0259	0.00443	0.01643	0.00780	*

b)* An. funestus*							
Populations	Punguene	Ribangua	3 de Fevereiro	Bobole	Macia	Palmeira 1°/2°/4° Bairro	Palmeira 7° Bairro Riverside
Punguene	*						
Ribangua	0.00246	*					
3 de Fevereiro	0.00466	0.00744	*				
Bobole	0.01394	0.01127	0.01477	*			
Macia	0.01148	0.00578	**0.02480**	0.00892	*		
Palmeira 1°/2°/4° Bairro	0.02071	0.01513	**0.02818**	**0.04213**	**0.02970**	*	
Palmeira 7° Bairro Riverside	0.00347	− 0.00457	0.00753	0.01307	0.00781	0.00843	*

Pairwise comparison of populations where a) *An. arabiensis* and b) *An. funestus* were collected to generate F_ST_ values. Statistically significant F_ST_ values are in bold (P < 0.05). P-values obtained after 10,000 permutations

The genetic structure variation among populations, among individuals within populations and within individuals of *An. arabiensis* and *An. funestus s.s.* were assessed across respective six loci by AMOVA (Additional file 1: Table S7). For both species, greater than 99% of the total genetic diversity was partitioned within populations, indicating no genetic structure.

An estimate of gene flow, N_m_, was calculated based on observed F_ST_ values for different populations of *An. arabiensis* and *An. funestus s.s.*. A moderate to high amount of gene flow was observed among the five populations of *An. arabiensis*, 9.4 to 56.2 migrants per generation, and among the ten populations of *An. funestus s.s.,* 5.7 to 71.8 migrants per generation (Additional file 1: Table S8). No significant correlation was observed between estimated number of migrants by distance (Additional file 1: Figure S3).

Bayesian clustering analysis did not reveal population structure for hypothetical *An. arabiensis* or *An. funestus s.s.* population clusters. The estimates for the posterior probability of Ln Pr(X|K) were inconclusive for *An. arabiensis*, where the value of K that showed the highest Ln Pr(X|K) followed by a plateau with increasing K was never reached; The posterior probability estimates were erratic for *An. funestus* as the value of K increased (Additional file 1: Fig. S4). Additionally, the proportions of each individual population of *An. arabiensis* or *An. funestus s.s.* assigned to cluster K were roughly symmetrical (~ 1/K in each population). These results indicate that the likelihood of an individual mosquito belonging to cluster K are unreliable and not clearly assigned to any one group for both *Anopheles* species.

## Discussion

Surveillance of malaria vectors encompasses a wide range of activities, from characterizing geographical distribution to investigating population structure. In this study, an initial investigation of insecticide resistance in the main malaria vectors, *An. funestus s.s.* and *An. arabiensis*, in and around Manhiça district revealed marked differences. Whereas *An. arabiensis* was fully susceptible to 1 × deltamethrin across most study collection sites, *An. funestus s.s.* showed a considerable range of 1 × deltamethrin resistance. Molecular analysis of genes that confer pyrethroid resistance, *kdr* for *An. arabiensis* and *CYP6P9a* in *An. funestus s.s.*, further supported this phenotypic evidence with 99% homozygous susceptible *kdr* in *An. arabiensis* and over 98% homozygous or heterozygous resistant *CYP6P9a* in *An. funestus s.s.*. The high level of *CYP6P9a* resistance in *An. funestus s.s.* plays an important role in the moderate to high phenotypic resistance observed. It is important to note that known insecticide resistance markers are only the tip of the iceberg. It is highly likely that there are other, currently undetected, genetic mutations underlying the observed variations in resistance in this study [36]. Previous genetic structure analysis of *An. funestus s.s.* in Zambia, Malawi, and Mozambique showed that barriers to gene flow can play a major role in the underlying genetic differences and varied phenotypic resistance profiles as well as in elucidating resistance mechanisms [9]. Alternatively, possible differences in the age distribution of mosquitoes across the collection sites may have contributed to the heterogeneity in resistance, which was not controlled for in this study [68, 69]. These initial findings led to the examination of population structure of *An. funestus s.s.* and *An. arabiensis* to characterize gene flow and its role, if any, in the spread of genes that confer insecticide resistance and to understand its implications for vector control interventions in Manhiça district and surrounding areas.

A moderate level of genetic differentiation was observed across the six loci each for *An. arabiensis* and *An. funestus s.s.* as evidenced by highly polymorphic loci, significant departures from Hardy–Weinberg Equilibrium, and moderate inbreeding coefficients (F_IS_). The results are consistent with previously reported genetic differentiation of *An. arabiensis* and *An. funestus s.s.* in eastern and southern Africa, including some areas in Mozambique [20–22, 60, 70–76]. Linkage disequilibrium revealed no linked loci in *An. arabiensis* but was high (33%) in *An. funestus s.s.*, suggesting possible population structure for this species across the study collection sites. Although moderate levels of genetic differentiation can be the result of geographic barriers to gene flow, that is likely not the case in this study area where there are no such significant features or large distances separating populations. However, it is noteworthy to point out that Ilha Josina is surrounded by swamps and inaccessible during the rainy season. There are many factors influencing the genetic differentiation in both *Anopheles* species such as null alleles or grouping of gene pools (Wahlund effect), which may underestimate heterozygosity or indicate population substructure, respectively. Inbreeding or nonrandom mating may also lead to heterozygote deficits. Additional factors may be spatial pooling from different houses or foci within houses and/or temporal pooling over a 2-month sampling period [77]. Genetic drift or epistatic natural selection, as well as environmental changes such as urbanization and global warming, can also have a substantial effect on the spread of genes that confer insecticide resistance and warrants further investigation [77].

This study successfully amplified six microsatellite loci for each *Anopheles* species, but is lower than several other *Anopheles* population genetics studies that use at least ten loci [20, 22, 24, 71, 76, 78]. By using fewer microsatellite loci, bias may be introduced due to selection acting on one or more loci, null alleles, and/or amplification failure, leading to a false impression of genetic differentiation across the study populations. For *An. arabiensis*, this weak amplification may also be due to limited usefulness of microsatellite markers designed for its sibling species *An. gambiae s.s.*, which has been previously reported for another sibling species, *An. melas* [79]. On the other hand, specimens of both *An. arabiensis* and *An. funestus s.s.* had been stored for 3 years and had undergone extensive transport prior to handling and use for laboratory assays, which may have affected specimen integrity and limited successful microsatellite amplification of several loci.

Further investigation of population pairwise comparisons (F_ST_) and the analysis of molecular variance (AMOVA) confirmed that there is no population structure for *An. arabiensis* and *An. funestus s.s.* in the study collection area. The low F_ST_ values observed in this study are consistent with other population genetics study for both *Anopheles* species [9, 20, 24, 25, 73, 75, 80–82]. The AMOVA results in this study area indicate that greater than 99% of the genetic variation is maintained within both *Anopheles* species populations, supporting the lack of genetic structure, and suggesting each *Anopheles* species as a single panmictic population in the study area. This genetic variance within populations is higher but consistent with recent findings by Kaddumukasa et al. [22] for *An. funestus s.s.* Similarly, the estimate for the exchange of genes among populations, also known as N_m_, has been reported to be between 3–101 for *An. arabiensis* and 6–483 for *An. funestus s.s.*, depending on the geographical scale surveyed [20–22, 70, 73, 83]. The N_m_ values for *An. funestus s.s.* of 5.7–101.4 and *An. arabiensis* of 9.4–56.2 in this study are similar, although given the relatively short distances between populations, the lower N_m_ values for *An. funestus s.s.* are particularly interesting. Overall, over these short geographical distances, no significant correlation was found between migration rates and distance between locations (Additional file 1: Fig. S1, *An. funestus s.s.* p = 0.13; *An. arabiensis* p = 0.11). In combination with the moderate genetic differentiation and high linkage disequilibrium recorded for *An. funestus s.s.*, there may be sub-populations that mostly interbreed but overlap, which could explain the phenotypic variation observed in deltamethrin resistance. Finally, for both *Anopheles* species, *Structure* analysis showed repeatedly inconsistent run results and no clear assignment to cluster K such that genetic structure could not be determined. In combination with the F_ST_, AMOVA, and N_m,_ the *Structure* results provide strong evidence that there is no population structure and that both *An. arabiensis* and *An. funestus s.s.* mosquitoes on this small geographical area of maximum 70 km radius are highly admixed.

To date, this is the first investigation of fine scale population genetic structure in Southern Mozambique, an area targeted for malaria elimination [84]. Compared to *An. gambiae s.s.* and *An. funestus s.s.*, there have been few population genetic studies of *An. arabiensis*, which is surprising given the remarkable plasticity of this species and consequences for malaria transmission [25, 70, 78, 83]. Donnelly and Townson reported in 2000 genetically distinct populations of *An. arabiensis* in two neighbouring villages in southern Mozambique, which we did not observe in our study [17]. Their result may be very specific to that location or time, stochastic effects resulting from small sample size, or perhaps our study did not have the power to detect these differences, despite having similar mean number of alleles observed in the area.

The high gene flow among populations of *An. arabiensis* and *An. funestus s.s.* in the study area cannot explain the observed heterogeneity in insecticide resistance to 1 × deltamethrin, particularly in *An. funestus s.s.* which showed a substantial and variable phenotypic resistance profile. Differential insecticidal pressure could be selecting for temporary spatial differences across these different sites prior to migration breaking down differences, though there is no data regarding the level of insecticide pressure across these different sites. There has been a particular effort in the Magude district to eliminate malaria between 2015 and 2018 [84] including annual rounds of IRS with DDT and pirimiphos-methyl, and pyrethroid-only long-lasting insecticide treated nets (LLINs). Yet, surprisingly, the highest overall level of pyrethroid susceptibility was seen in the two villages in this elimination-targeted area, though this is based on low sample numbers due to the eradication efforts. Alternatively, phenotypic differences between sites could also in part be explained by variation in time and day of conducting the CDC bottle bioassay rather than true phenotypically different populations [85]. A recent study demonstrated that CDC bottle bioassays have a high level of variation in mortality measurements [86]. To rule out this option, in future studies CDC bottle bioassays should be conducted in the different areas on multiple days, which is rarely done, or alternative assays used such as the more consistent topical application bioassay [86]. Phenotypic resistance across sites could further be explained by environmental exposures that trigger epigenetic changes [87], microRNAs [88], and the composition of the mosquito microbiome [90].

The lack of population structure for both *Anopheles* species may be an advantage for this region, which is positioned for malaria elimination and includes the possibility of a transgenic mosquito release programme [76, 90]. The spread of such genes is predicted to be largely successful for both major malaria vectors.

## Conclusion

There was no evidence of population genetic structure of *An. arabiensis* and *An. funestus s.s.* in the 1650 square-kilometre area that was studied in southern Mozambique. Although moderate to high genetic differentiation was observed, almost all genetic diversity (> 99%) occurred within populations and other measures of gene flow suggest a single highly admixed population for each *Anopheles* species. Continued molecular surveillance of *An. arabiensis* and *An. funestus s.s.* population dynamics using additional polymorphic microsatellite loci or other molecular tools and focused sampling plan will be critical for assessing local changes in gene flow. The current results suggest that insecticide resistance genes, including future ones to novel active ingredients, may easily spread in the area, which can impact the efficacy of vector control strategies, but also that possible transgenic mosquito release programmes will likely not encounter any challenges in local expansion and coverage.

## Supplementary Information

**Additional file 1.** Supplemental file containing supplemental tables S1-S9 and supplemental figures S1-S4.

## Data Availability

The datasets used in this study are available from the corresponding author on reasonable request.
