# Supplementary material for: Fine-scale spatial distribution of deltamethrin resistance and population structure of Anopheles funestus and Anopheles arabiensis populations in Southern Mozambique
Source: Malar J. 2023 Mar 14;22:94. doi: 10.1186/s12936-023-04522-5 (PMC10010967; doi:10.1186/s12936-023-04522-5)
Supplement: Supplementary file 1 — Additional file 1. Supplemental file containing supplemental tables S1-S9 and supplemental figures S1-S4. [file 12936_2023_4522_MOESM1_ESM.pdf]

Supplemental information

Table S1. CDC bottle bioassay mortality data of 1x deltamethrin exposure across Manhica district. following 30 minute (diagnostic time) and 120 minute (end of assay) exposure of deltamethrin at 1x the diagnostic dose (12.5ug/bottle) and 5x the diagnostic dose (60ug/bottle). Following morphological identification, mortality data of *An. funestus s.s.* and *An. arabiensis* were calculated.

			All specimens		All specimens		*An. funestus s.s.*		*An. arabiensis*	
			**30mins mortality**		**120mins mortality**		**120mins mortality**		**120 mins mortality**	
	**Insecticide**	**concentration**	**exposed (%)**	**control (%)**	**exposed (%)**	**control (%)**	**exposed (%)**	**control (%)**	**exposed (%)**	**control (%)**
3 de Fevereiro	DM	1x	10.1 (n=89)	0 (n=17)	43.8 (n=89)	0 (n=17)	40.8 (n=76)	0 (n=14)	87.5 (n=8)	NA
	DM	5x	28.4 (n=81)	0 (n=22)	71.6 (n=81)	0 (n=22)	76.0 (n=50)	0 (n=19)	100 (n=3)	0 (n=1)
Bobole	DM	1x	8.5 (n=82)	0 (n=18)	19.5 (n=82)	0 (n=18)	15.2 (n=79)	0 (n=17)	100 (n=2)	NA
	DM	5x	28.6 (n=63)	0 (n=18)	85.7 (n=63)	0 (n=16)	79.5 (n=44)	0 (n=12)	82.4 (n=17)	0 (n=4)
Buna	DM	1x	3.9 (n=129)	0 (n=34)	6.2 (n=129)	0 (n=34)	4.1 (n=123)	0 (n=33)	100 (n=3)	NA
	DM	5x	4.4 (n=113)	0 (n=12)	7.1 (n=113)	0 (n=12)	2.8 (n=109)	0 (n=12)	100 (n=3)	NA
Chobela	DM	1x	85.0 (n=60)	0 (n=20)	100 (n=60)	0 (n=20)	100 (n=3)	NA	100 (n=50)	0 (n=18)
	DM	5x	99.1 (n=111)	0 (n=24)	99.1 (n=111)	0 (n=24)	92.3 (n=13)	0 (n=3)	100 (n=38)	0 (n=9)
Ilha Josina	DM	1x	88.8 (n=89)	0 (n=22)	91.0 (n=89)	0 (n=22)	53.3 (n=15)	0 (n=4)	98.5 (n=67)	0 (n=18)
	DM	5x	94.4 (n=89)	0 (n=15)	96.6 (n=89)	0 (n=15)	66.7 (n=9)	0 (n=2)	100 (n=73)	0 (n=11)
Macia	DM	1x	10.9 (n=128)	0 (n=29)	70.3 (n=128)	0 (n=29)	70.0 (n=120)	0 (n=27)	100 (n=5)	0 (n=2)
	DM	5x	21.8 (n=110)	0 (n=26)	77.3 (n=110)	0 (n=26)	77.4 (n=106)	0 (n=22)	100 (n=4)	0 (n=3)
Maragra Maciana	DM	1x	31.6 (n=19)	0 (n=9)	52.6 (n=19)	0 (n=9)	60.0 (n=20)	0 (n=4)	100 (n=1)	0 (n=2)
	DM	5x	37.5 (n=16)	0 (n=10)	75.0 (n=16)	0 (n=10)	NA	NA	NA	NA
Mulelemane	DM	1x	81.6 (n=76)	0 (n=13)	100 (n=76)	0 (n=13)	100 (n=1)	NA	100 (n=73)	0 (n=13)
	DM	5x	100 (n=70)	0 (n=17)	100 (n=70)	0 (n=17)	NA	NA	100 (n=69)	0 (n=18)
Palmeira 1°/2°/4° Bairro	DM	1x	7.9 (n=63)	0 (n=15)	38.1 (n=63)	0 (n=15)	47.9 (n=48)	0 (n=14)	100 (n=1)	0 (n=2)
	DM	5x	14.8 (n=61)	0 (n=15)	63.9 (n=61)	0 (n=15)	63.6 ((n=55)	0 (n=15)	NA	NA
Punguene	DM	1x	33.9 (n=165)	0 (n=50)	84.2 (n=165)	0 (n=50)	84.3 (n=121)	0 (n=44)	100 (n=12)	0 (n=3)
	DM	5x	50.3 (n=161)	0 (n=46)	84.5 (n=161)	0 (n=46)	87.7 (n=154)	0 (n=40)	100 (n=38)	0 (n=3)
Ribangua	DM	1x	18.3 (n=93)	0 (n=22)	44.1 (n=93)	0 (n=22)	39.0 (n=82)	0 (n=21)	100 (n=6)	NA
	DM	5x	82.3† (n=84)	5.9 (n=17)	93.7† (n=84)	5.9 (n=17)	100 (n=73)	7.1 (n=14)	100 (n=9)	0 (n=3)

DM=Deltamethrin; † Abbott's formula applied

Table S2. CDC bottle bioassay mortality data of different barrios in the town of Palmeira following 30-minute (diagnostic time) and 120-minute (end of assay) exposure of deltamethrin at 1x (12.5ug/bottle) and 5x (60ug/bottle) the diagnostic dose. Following morphological identification, mortality data of *An funestus s.s.* and *An. arabiensis* were calculated.

			All specimens		All specimens		*An. funestus*		*An. arabiensis*	
			**30mins mortality**		**120mins mortality**		**120mins mortality**		**120 mins mortality**	
**Neighborhood**	**Insecticide**	**concentration**	**exposed**	**control**	**exposed**	**control**	**exposed**	**control**	**exposed**	**control**
1°,2°, 4° Bairro	DM	1x	7.9 (n=63)	0 (n=15)	38.1 (n=63)	0 (n=15)	47.9 (n=48)	0 (n=14)	100 (n=1)	0 (n=2)
	DM	5x	14.8 (n=61)	0 (n=15)	63.9 (n=61)	0 (n=15)	63.6 ((n=55)	0 (n=15)	NA	NA
3° Bairro A – Roadside	DM	1x	9.6 (n=115)	2.4 (n=42)	14.8 (n=115)	2.4 (n=42)	16.2 (n=117)	0 (n=41)	100 (n=3)	NA
	DM	5x	22.6 (n=53)	0 (n=18)	52.8 (n=53)	0 (n=18)	55.1 (n=49)	0 (n=11)	NA	NA
3° Bairro A – Riverside	DM	1x	10.1 (n=79)	0 (n=19)	24.1 (n=79)	0 (n=19)	22.4 (n=67)	0 (n=19)	NA	NA
	DM	5x	28.8 (n=66)	0 (n=21)	66.7 (n=66)	0 (n=21)	66.7 (n=60)	0 (n=21)	NA	NA
3° Bairro B	DM	1x	10.4 (n=96)	NA	29.2 (n=96)	NA	26.6 (n=94)	NA	100 (n=2)	NA
	DM	5x	16.9 (n=77)	0 (n=22)	41.6 (n=77)	0 (n=22)	52.7 (n=74)	0 (n=23)	100 (n=1)	NA
3° Bairro C	DM	1x	9.2† (n=23)	4.2 (n=24)	36.4† (n=23)	4.2 (n=24)	22.2 (n=18)	0 (n=21)	100 (n=5)	0 (n=2)
	DM	5x	NA	NA	NA	NA	NA	NA	NA	NA
6° Bairro – Roadside	DM	1x	17.7 (n=62)	NA	87.1 (n=62)	NA	86.2 (n=58)	NA	100 (n=2)	NA
	DM	5x	5.0 (n=180)	0 (n=41)	21.7 (n=180)	0 (n=41)	22.0 (n=132)	0 (n=40)	100 (n=3)	0 (n=2)
6° Bairro – Riverside	DM	1x	6.4 (n=63)	0 (n=23)	61.9 (n=63)	0 (n=23)	61.3 (n=62)	0 (n=23)	NA	NA
	DM	5x	24.4 (n=82)	NA	84.2 (n=82)	NA	84.3 (n=51)	NA	100 (n=1)	NA
7° Bairro A – Roadside	DM	1x	6.9 (n=101)	NA	11.9 (n=101)	NA	9.3 (n=97)	NA	100 (n=2)	NA
	DM	5x	29.2 (n=65)	0 (n=16)	72.3 (n=65)	0 (n=16)	71.0 (n=62)	0 (n=15)	NA	NA
7° Bairro A – Riverside	DM	1x	5.6 (n=107)	0 (n=25)	50.5 (n=107)	0 (n=25)	60.2 (n=103)	0 (n=26)	100 (n=4)	NA
	DM	5x	13.3 (n=83)	0 (n=16)	63.9 (n=83)	0 (n=16)	58.2 (n=79)	0 (n=15)	100 (n=1)	NA

Table S3. CDC bottle bioassay mortality data across the district following 30-minute (diagnostic time) and 120-minute (end of assay) exposure of bendiocarb at 1x the diagnostic dose (12.5ug/bottle). Following morphological identification, mortality data of *An. funestus s.s.* and *An. arabiensis* were calculated.

			All specimens		All specimens		*An. funestus s.s.*		*An. arabiensis*	
			**30mins mortality**		**120mins mortality**		**120mins mortality**		**120 mins mortality**	
	**Insecticide**	**concentration**	**exposed**	**control**	**exposed**	**control**	**exposed**	**control**	**exposed**	**control**
Chobela	BC	1x	100 (n=90)	0 (n=17)	100 (n=90)	0 (n=17)	100 (n=17)	0 (n=3)	100 (n=43)	0 (n=13)
Mulelemane	BC	1x	100 (n=90)	8.0 (n=25)	100 (n=90)	4.0 (n=25)	100 (n=1)	0 (n=1)	100 (n=89)	4.5 (n=22)
Palmeira<br>7° Bairro Roadside	BC	1x	99 (n=104)	0 (n=19)	100 (n=104)	0 (n=19)	100 (n=105)	0 (n=19)	NA	NA
Ribangua	BC	1x	100 (n=73)	0 (n=17)	100 (n=73)	0 (n=17)	100 (n=66)	0 (n=17)	100 (n=7)	NA

Table S4. Genetic variability of 6 microsatellite loci in *Anopheles arabiensis* from Southern Mozambique. Number of alleles (N_{allele}), observed (H_o), expected heterozygosity (H_e), and inbreeding coefficient (F_{IS}) for each loci in *An. arabiensis* collected from five populations. Bold H_e indicate significant deviation ($P < 0.00197$) from Hardy-Weinberg equilibrium.

Locus		Populations					
		**Chobela (N=33)**	**Ilha Josina (N=34)**	**Magude-Mulelemani (N=33)**	**Palmeira 1°/2°/4° Bairro (N=30)**	**Punguene (N=29)**	**Overall (n=159)**
**AGXH25**	N_{allele}	5	6	6	4	5	6
	H_o	0.12121	0.11765	0.09091	0.06897	0.13793	0.10759
	H_e	**0.66993**	**0.57682**	**0.58695**	**0.41077**	**0.51724**	**0.56645**
	F_{IS}	0.82135	0.79847	0.84713	0.83456	0.73678	0.80936
**AG2H85**	N_{allele}	6	4	4	4	3	6
	H_o	0.42424	0.45455	0.27273	0.43333	0.31034	0.37975
	H_e	**0.69790**	**0.58415**	**0.64755**	**0.68079**	**0.66606**	**0.66050**
	F_{IS}	0.39582	0.22456	0.58261	0.36745	0.53846	0.42376
**AG2H164**	N_{allele}	7	7	9	7	7	12
	H_o	0.45455	0.52941	0.72727	0.43333	0.62069	0.55346
	H_e	0.52121	0.69271	0.72914	0.55028	0.59952	0.62901
	F_{IS}	0.12965	0.23846	0.00260	0.21540	-0.03597	0.11010
**AG3H127**	N_{allele}	Monomorphic	2	Monomorphic			2
	H_o		0.00000				0.00000
	H_e		**0.16783**				**0.03784**
	F_{IS}		1.00000				1.00000
**AGXH100**	N_{allele}	6	7	7	7	7	7
	H_o	0.54545	0.65625	0.66667	0.70000	0.60714	0.63462
	H_e	0.66107	0.65675	0.71329	0.77401	0.62468	0.68866
	F_{IS}	0.17714	0.00077	0.06631	0.09711	0.02857	0.07663
**AG3H249**	N_{allele}	14	11	15	9	9	18
	H_o	0.64516	0.57576	0.61290	0.56667	0.64286	0.60784
	H_e	0.74352	0.72448	0.79376	0.63898	0.74935	**0.73215**
	F_{IS}	0.13420	0.20782	0.23077	0.11490	0.14437	0.16987
**Mean across loci**	N_{allele}	7.6	6.2	8.2	6.2	6.2	8.5
	H_o	0.43812	0.38894	0.47410	0.44046	0.46379	0.38054
	H_e	0.65873	0.56712	0.69414	0.61097	0.63137	0.55244
	F_{IS}	0.33226	0.30441	0.31231	0.27678	0.26036	0.29934

Table S5. Genetic variability of 6 microsatellite loci in *Anopheles funestus s.s.* from Southern Mozambique. Number of alleles (N_{allele}), observed (H_o), expected heterozygosity (H_e), and inbreeding coefficient (F_{IS}) for each loci in *An. funestus* collected from five populations. Bold H_e indicate significant deviation (P < 0.00122) from Hardy-Weinberg equilibrium.

		Populations							
**Locus**		**Punguene (n=45)**	**Ribangua (n=49)**	**3 de Fevereiro (n=53)**	**Bobole (n=33)**	**Macia (n=48)**	**Palmeira 1°/2°/4° Bairro (n=38)**	**Palmeira 7° Bairro Riverside (n=58)**	**Overall (n=324)**
**FunG**	N_{allele}	12	11	10	10	13	10	11	14
	H_o	0.67442	0.62222	0.83019	0.74194	0.77778	0.78378	0.80000	0.75081
	H_e	**0.90315**	**0.88190**	0.88266	**0.87996**	**0.90637**	0.81081	**0.86722**	**0.89508**
	F_{IS}	0.25550	0.29680	0.05998	0.15905	0.14325	0.03378	0.07818	0.14555
**FunO**	N_{allele}	4	5	5	4	5	3	5	10
	H_o	0.50000	0.38298	0.48148	0.40000	0.47826	0.48485	0.33929	0.43110
	H_e	0.55669	**0.57630**	0.58001	0.54407	0.56355	0.57576	0.58526	**0.57114**
	F_{IS}	0.10289	0.33786	0.17258	0.26814	0.15276	0.15997	0.42249	0.24581
**AFND6**	N_{allele}	11	12	11	11	13	9	9	19
	H_o	0.46341	0.47727	0.44000	0.50000	0.40909	0.36364	0.45098	0.44643
	H_e	**0.86871**	**0.84065**	**0.82808**	**0.82208**	**0.83438**	**0.87844**	**0.84547**	**0.85004**
	F_{IS}	0.46964	0.43510	0.47118	0.39617	0.51259	0.59174	0.46907	0.47391
**AFND32**	N_{allele}	10	12	8	8	9	7	10	13
	H_o	0.40000	0.57143	0.49057	0.54545	0.52083	0.44737	0.51724	0.50000
	H_e	**0.81448**	**0.84999**	**0.78365**	**0.82517**	**0.80570**	**0.80175**	**0.83763**	**0.82604**
	F_{IS}	0.51171	0.33001	0.37624	0.34247	0.35599	0.44533	0.38456	0.39080
**AFND40**	N_{allele}	8	8	7	7	6	6	6	9
	H_o	0.26471	0.17073	0.23077	0.26667	0.29032	0.21053	0.25000	0.23741
	H_e	**0.82002**	**0.84372**	**0.77819**	**0.78305**	**0.81227**	**0.82000**	**0.78771**	**0.81571**
	F_{IS}	0.68047	0.79964	0.70549	0.66328	0.64637	0.74581	0.68474	0.70766
**FunD**	N_{allele}	19	16	18	13	20	17	20	30
	H_o	0.79545	0.81633	0.84314	0.81818	0.85417	0.76316	0.79310	0.81308
	H_e	0.90596	0.91668	0.90526	0.88904	0.89803	0.90000	0.90690	**0.90485**
	F_{IS}	0.12322	0.11049	0.06926	0.08085	0.04933	0.15379	0.12644	0.10161
**Mean across loci**	N_{allele}	10.7	10.7	9.8	8.8	11.0	8.7	10.2	15.8
	H_o	0.51633	0.50683	0.55269	0.54537	0.55508	0.50889	0.52510	0.52980
	H_e	0.81150	0.81821	0.79298	0.79056	0.80338	0.79779	0.80503	0.81048
	F_{IS}	0.27470	0.22116	0.14982	0.17466	0.15967	0.20316	0.18157	0.19390

Table S6. Linkage disequilibrium of six microsatellite loci for *An. funestus s.s.*. Statistically significant pairwise comparisons (P < 0.05) are indicated by "+" in black boxes with the exact P-value in bold. Linkage disequilibrium that is not significant is indicated with a "-" (P > 0.00341).

	**FunG**	**FunO**	**AFND6**	**AFND32**	**AFND40**	**FunD**
**FunG**	*					
**FunO**	**+ (P= 0.00000)**	*				
**AFND6**	-	**+ (P= 0.00000)**	*			
**AFND32**	-	-	-	*		
**AFND40**	-	**+ (P= 0.012)**	**+ (P=0.006)**	-	*	
**FunD**	**+ (P= 0.00000)**	-	-	-	-	*

Table S7. Analysis of molecular variance (AMOVA) of six microsatellite loci each in the *An. arabiensis* and *An. funestus* populations.

a) An. arabiensis

Populations	Sum of squares	Variance components	Percentage variation
**Among populations**	11.296	0.01053	0.63405
**Among individuals within populations**	327.771	0.50886	30.63518
**Within individuals**	178.500	1.14163	68.78077

b) An. funestus s.s.

Populations	Sum of squares	Variance components	Percentage variation
**Among populations**	28.291	0.01694	0.69575
**Among individuals within populations**	946.363	0.82898	34.03970
**Within individuals**	484.000	1.58941	65.26455

Table S8. Estimated number of migrants among a) *An. arabiensis* and b) *An. funestus* populations.

a) An. arabiensis

Populations	Punguene	Magude-Mulelemani	Palmeira 1°/2°/4° Bairro	Chobela	Ilha Josina
**Punguene**	*				
**Magude-Mulelemani**	47.3	*			
**Palmeira** 1°/2°/4° Bairro	16.9	24.8	*		
**Chobela**	26.5	30.4	23.7	*	
**Ilha Josina**	9.4	56.2	15.0	31.8	*

b) An. funestus s.s.

Populations	Punguene	Ribangua	3 de Fevereiro	Bobole	Macia	Palmeira 1°/2°/4° Bairro	Palmeira 7° Bairro Riverside
**Punguene**	*						
**Ribangua**	101.4	*					
**3 de Fevereiro**	53.4	33.4	*				
**Bobole**	17.7	21.9	16.7	*			
**Macia**	21.5	43.0	9.8	27.8	*		
**Palmeira** 1°/2°/4° Bairro	11.8	16.3	8.6	5.7	8.2	*	
**Palmeira** 7° Bairro Riverside	71.8	54.5	33.0	18.7	31.8	29.4	*

Table S9. Microsatellite primer sequences and primer labels for microsatellite analysis of a) Anopheles arabiensis and b) An. funestus.

a) An. arabiensis

Microsatellite	Primer	Primer sequence (5' to 3')
AGXH25	AGXH25-F	/56-FAM/ GCC GAA AAC ATT CCA ACA GG
	AGXH25-R	CAG TTA TGT CGG CAT GCT AC
AG2H85	AG2H85-F	/5HEX/ ATT TAT CAT ACG GCG CCC AC
	AG2H85-R	TTG AAA GGT TGC AAC GAG CGC G
AG2H164	AG2H164-F	/56-FAM/ GTG GTA CCT CTG TCA TAC CC
	AG2H164-R	ACA ACA AAA GGC ACC GCA GC
AG3H127	AG3H127-F	/5HEX/ CCT CTA ACT CGA TTA CCG TG
	AG3H127-R	GTC AGG GAA TTG GAA AGA GC
AGXH100	AGXH100-F	/56-FAM/ AGA AAG GAA ATG TAA CGC GG
	AGHX100-R	CTT TCA TCT TGG CTG GCT GC
AG3H249	AG3H249F	/5HEX/ATG TTC CGC ACT TCC GAC AC
	AG3H249-R	GCG AGC TAC AAC AAT GGA GC

b) An. funestus

Microsatellite	Primer	Primer sequence (5' to 3')
FunO	FunO-F	/5HEX/ GCA CAC ATT TCA GGC AGC
	FunO-R	GCC CAC ATT CTG CAC CTT
FunG	FunG	/56-FAM/ GAG CAA GCA GCT TAC TGC AC
	FunG-R	ACG TTC AGT GCA CAT CAA TG
AFND6	AFND6-F	/5HEX/ GCT TCT TCT CCC CTA ATC TG
	AFND6-R	TCC TGC TTT TTA GTT TGT CG
AFND32	AFND32-F	/56-FAM/ GAA GCA TTT TGG GTT AGA CTC
	AFND32-R	GCA GTT GTT TAC CTT TCA CTG
FunD	FunD-F	/5HEX/ GCT AAC TAC TCC GAA GCG CT
	FunD-R	GAT CGC AAA ACT TCC GGT T
AFND40	AFND40-F	/56-FAM/ ATT CAT CCT GTG ATG CTT TG
	AFND40-R	AGG CTC TTC TTT GCA CTG T

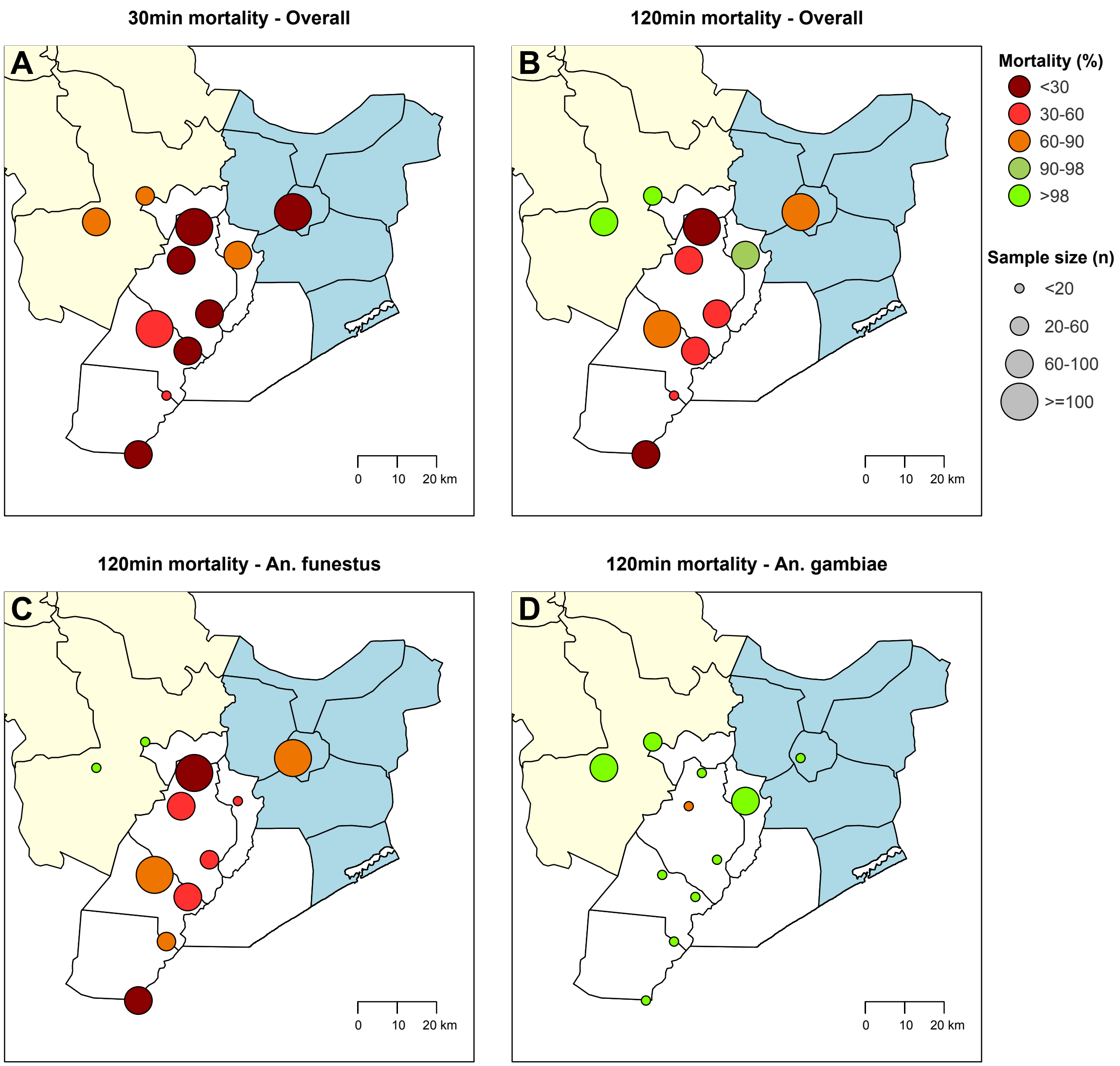

Figure S1. Spatial distribution of mortality of *Anopheles* spp populations in CDC bottle bioassays at 1x deltamethrin following a 30min exposure (A) and 120min exposure (B). Mortality following 120min exposure after mosquito identification is shown for *An. funestus s.s.* (C) and *An. gambiae s.l.* (predominantly *An. arabiensis*) (D). Levels of mortality are indicated by different colors ranging from less than 30% to over 98%, sample size of exposed mosquitoes in the bottles is demonstrated by the size of the circle. Palmeira data is from neighborhood 1°/2°/4° Bairro.

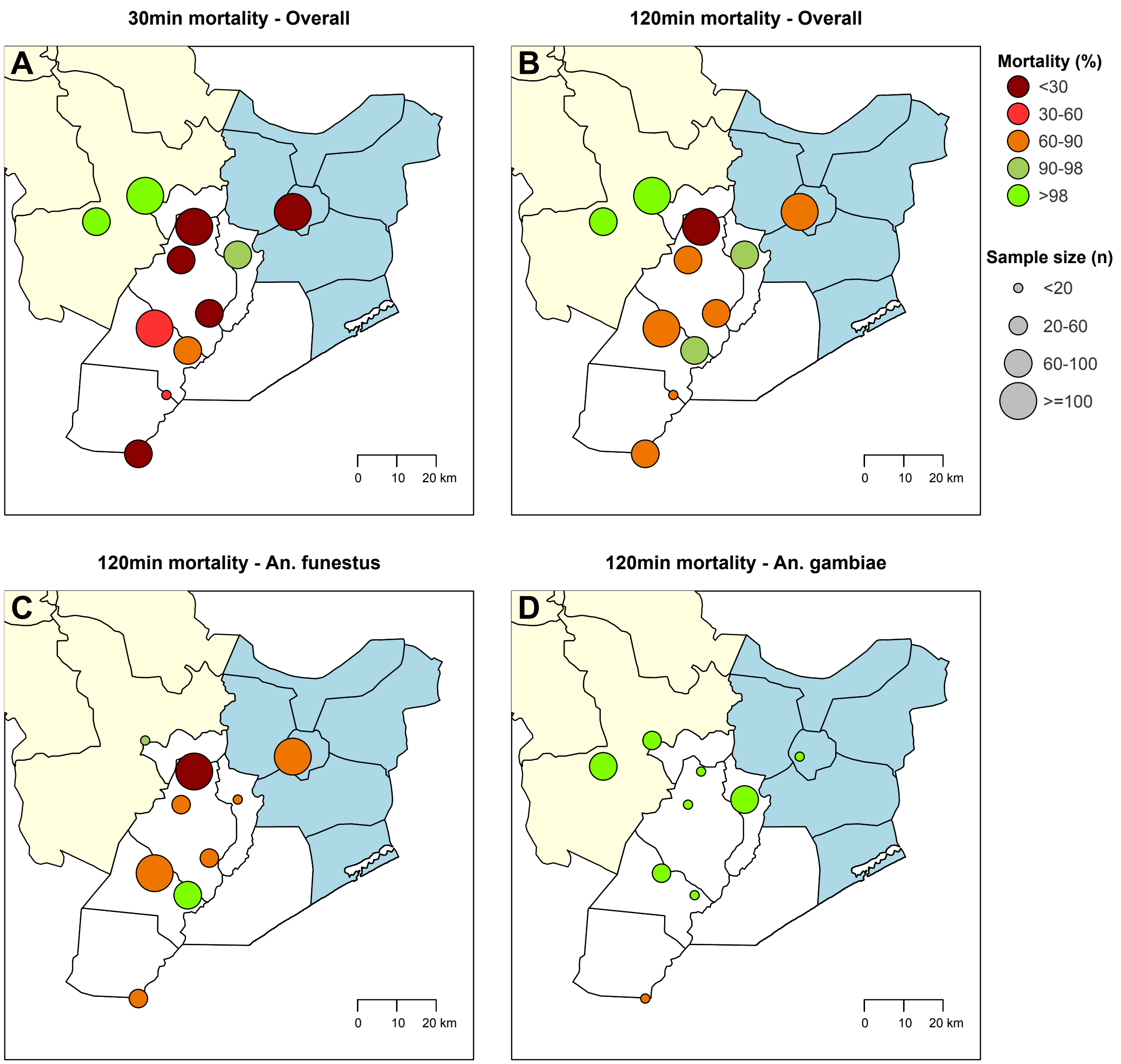

Figure S2. Spatial distribution of mortality of *Anopheles* spp populations in CDC bottle bioassays at 5x deltamethrin following a 30min exposure (A) and 120min exposure (B). Mortality following 120min exposure after mosquito identification is shown for *An. funestus s.s.* (C) and *An. gambiae s.l.* (predominantly *An. arabiensis*) (D). Levels of mortality are indicated by different colors ranging from less than 30% to over 98%, sample size of exposed mosquitoes in the bottles is demonstrated by the size of the circle. Palmeira data is from neighborhood 1°/2°/4° Bairro.

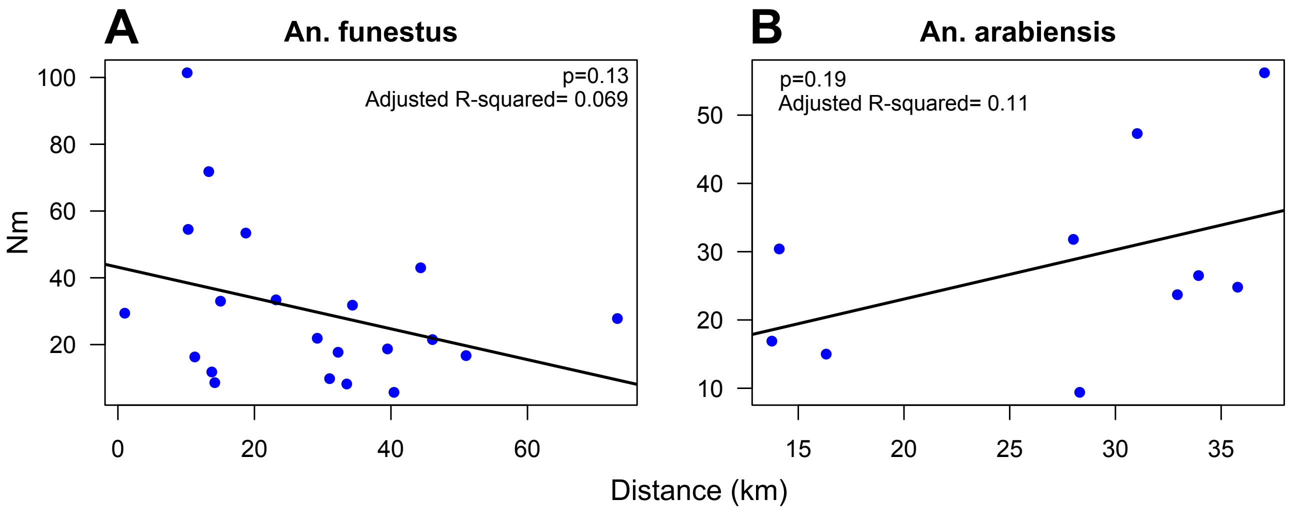

Figure S3. Estimated number of migrants per generation between two locations by distance (in kilometers) for *An. funestus s.s.* (A) and *An. arabiensis* (B). Trendline shows linear regression and p-value shows level of significance for the impact of distance.

a) An. arabiensis

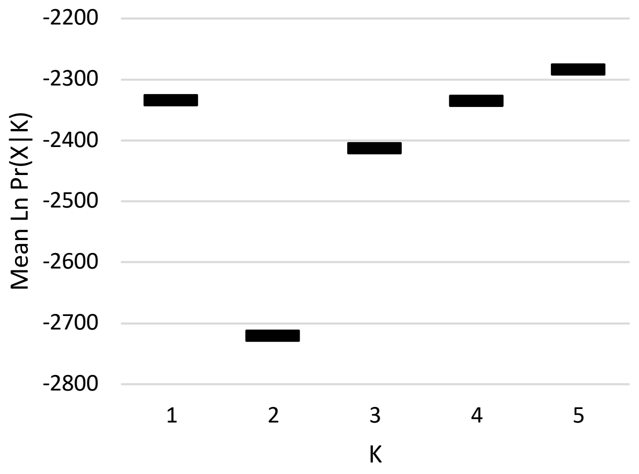

b) An. funestus

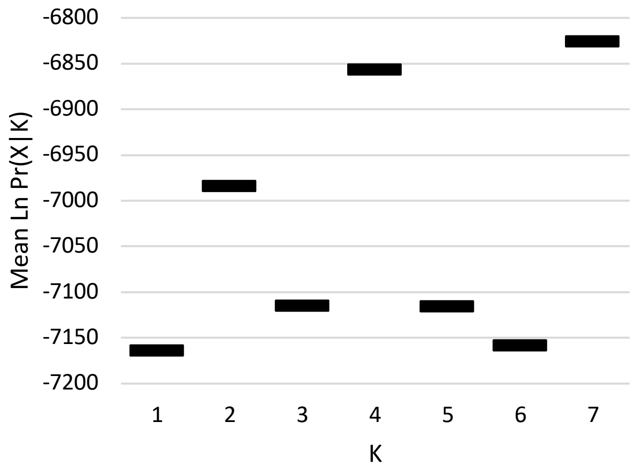

Figure S4. Estimated population structure from STRUCTURE analyses for a) *Anopheles arabiensis* and b) *An. funestus*. The mean posterior probability LnPr(X|K) based on 5 replicated runs for each clusters K=1 to K=5 for *An. arabiensis* and K=1 to K=7 for *An. funestus*.
